# A Qualitative Analysis of a USB Camera for AGV Control

**DOI:** 10.3390/s19194111

**Published:** 2019-09-23

**Authors:** Diogo Puppim de Oliveira, Wallace Pereira Neves dos Reis, Orides Morandin Junior

**Affiliations:** 1 TEAR - Laboratório de Estratégias de Automação e Robótica, Department of Computing, Federal University of São Carlos, São Carlos, SP 13565-905, Brazil; wallace.reis@ifrj.edu.br (W.P.N.d.R.); orides@ufscar.br (O.M.J.); 2 Federal Institute of Science, Education and Technology of Rio de Janeiro - IFRJ, campus Volta Redonda, Volta Redonda, RJ 27215-350, Brazil

**Keywords:** automated guided vehicles, USB camera, control system, position sensor, image processing, computer vision

## Abstract

The increasing use of Automated Guided Vehicles (AGV) in the industry points to a search for better techniques and technologies to adapt to market requirements. Proper position control and movement give an AGV greater movement accuracy and greater lateral oscillations stability and vibration. It leads to smaller corridors and leaner plants, to more relaxed shipment devices, and to greater safety in the transport of fragile loads, for instance. AGV control techniques are not new, but new sensors’ applications are possible, such as USB cameras. In this sense, it is necessary to ensure the sensor is adequate to control system requirements. This work addresses AGVs driven by passive floor demarcations. It presents a qualitative analysis of a USB camera as sensors for AGV control, not yet a common industrial application. We performed the experiments with a small AGV prototype on an eight-shaped lane, varying both camera parameters and AGV parameters, such as linear speed. The AGV uses a USB camera with different image processing settings—different morphological filters structuring elements shapes and sizes, and three different image resolutions—to analyze the factors that affect line detection and control processing. This paper’s main contribution is a qualitative and quantitative analysis for the different sensor configurations. In addition, it discusses the influence sources on camera image as a position sensor. Furthermore, the experiments confirm sensor pertinence for the proposed control system.

## 1. Introduction

Today’s manufacturing industries demand efficient and flexible solutions in automation and robotics to remain competitive in such a globalized market [1]. These intelligent manufacturing and logistics systems have contributed to mobile robotics improvement as a highly flexible solution [2]. An Automated Guided Vehicle (AGV) is a mobile robot/vehicle to carry materials in industrial environments. It receives high-level instructions according to factory plant needs and uses a guide path as displacement itinerary to execute material collection or distribution up to the determined point [3].

There are several types of sensors used to guide AGVs such as electromagnetic and optical [4]. About the guidance technology, Fedorko et al. [5] state that a “popular and affordable version is still a version using a magnetic tape”. The commercial magnetic sensors feature 1 mm resolution and a refresh rate of 100 Hz [6]. However, magnetic or RFID-guided systems – using Radio Frequency Identification tags – require pre-planned installations of their trajectories, making it difficult to change routes, reducing the flexibility of the manufacturing environment [7]. Intelligent AGVs, though, use computer vision systems, aided by other sensors, to work with more flexible paths. Commercial AGVs with indoor navigation systems do not use visual orientation as much as magnetic orientation due to a lack of evidence and data that convinces industry professionals of their benefits. However, the visual guidance system provides a simple, high-performance, and low-cost control. It allows the AGV to recognize the different crossroads, choose its next path and trace its guide tracks reaching the final destination [8].

Thus, to summarize the line following problem, the AGV control system must minimize the distance and the deviation angle related to the guideline, considering a fixed reference point in AGV structure. The AGV overall system needs to attain to performance indicators, compared among different system settings. This work considers the position exactness, meaning how close two sequential measures are and the AGV ability to return to the guideline. The question is how the sensor characteristics directly affect the indicator.

The article aims to evaluate the USB camera use as a sensor in the AGV position/path control loop. For these controls, the acquired images need pre-processing, which requires dedication of the AGV processor. However, this processing cannot degrade the control loop performance. The image processing elapsed time cannot affect the sampling time of the digital controller. The work also proposes to assess the impact of image resolution, image processing parameters, and frame per second (fps) rate on position/path control. Besides the image processing characteristics variation, the experiments varied the physical characteristics of the robot like linear speed. These changes estimate the sensor response critical scenarios among different characteristics’ combinations in the experiments from the controller’s point of view.

The proposal is to appraise the sensor characteristics necessary for an AGV to use an USB camera as a position sensor suitable to its control loop, i.e., meeting the position control needs, and not degrading it. We carried out experiments with an affordable and low-cost USB RGB (Red-Green-Blue) Camera, Microsoft LifeCam Webcam (Microsoft China Co. Ltd., China), and a portable computer to evaluate qualitatively and quantitatively the impact of the cited variations on the control loop.

The paper organization is as follows: Section 2 introduces related work online following AGVs and the use of an USB camera as a position sensor in AGVs. Then, the overall system and proposed approach are respectively described in detail in Section 3. Section 4 presents the experiments and its results, while Section 5 discusses the experimental results. Finally, Section 6 provides the work conclusions.

## 2. Related Works

Cawood and Gorlach [9] present an Automated Guided Cart (AGC), “an AGV that provides material handling capability with a low level of control and complexity”. General Motors South Africa requested the AGC development. The work focuses on navigation and locomotion of the AGC, proposing an infrared sensor line-following system. The authors claim “simpler alternatives [for line-following] lie in using either several infrared (IR) or induction sensors”. The proposed AGC used a line of three or five IR sensors to detect the lateral movement and correct the trajectory. The results show that the AGC movement of each sensor setup impacts RFID stop markers’ detection, a design requirement.

Henebrey and Gorlach [10] present an enhancement in an AGC “used for material handling at the General Motors South Africa (GMSA) assembly plant in Struandale near Port Elizabeth”. The original system uses a magnetic tape and an MGS1600C magnetic guide sensor from Roboteq® (Scottsdale, AZ, USA). The authors propose a retrofitting in the motion system, keeping the original Programmable Logic Controller (PLC) and sensors, and changing the motors and its drivers. It requires new magnetic sensor calibration, due to velocity changes. The main problem was the overheating of drive motors after a long run and the authors solved it. The aforementioned works also show that the industries still use and research magnetic and IR guidance systems for AGVs.

Different systems can use a camera as a sensor. For instance, Wei et al. [11] present a sensor fusion in a collision avoidance system. The system uses a single RGB camera and a LiDAR to object detection and localization. Instead of a stereo vision system, the authors claim to require considerable computational processing, not to mention a difficulty with object depth estimation when it lacks pattern cues. The authors proposed a real-time collision avoidance system for industrial use. They tested it in different conditions.

The line-following approach using a vision system is not new, but it is not a depleted subject. Yu, Lou, and Wu [12] present a vision-based two-camera guided AGV structure and an embedded real-time processing system. The robot uses a frontal camera for prediction and a camera positioned in the vehicle center for positioning. In addition, the vehicle collects information from RFID tags along the track, “by which it identifies different working stations, parking stations, lane network nodes, and warehouses.” The proposed system presents an improvement in the speed of AGV and the accuracy of reading and compared them to the system proposed by [13].

Horan et al. [14] introduce OzTug, a “low-cost, robust and small in size” AGV. A USB 2.0 camera is responsible for acquiring images of a green guidance line in a 1280 × 1024 resolution at 25 frames per second. The image processing results in lateral deviation of the center of the detected line to the robot’s center of gravity and longitudinal offset measures. These values are the input variables of the fuzzy controller. The work presents three robot-load configurations and the simulation results from each of them, comparing the error area in square meters.

Wang et al. [15] present an enhanced vision navigation system using a Fuzzy Control Algorithm tested in an AGV for medical area use. The authors list the benefits of a vision navigation method as lower cost, easy installation, electromagnetic interference immunity, among others, compared to other industrial methods. The AGV detects the line with the camera and uses line information to calculate its coordinates in the environment using an inverse perspective transformation. They mount the camera in AGV lateral and ground lines from a corridor to the robot different from common applications where AGV moves above the line.

Liu et al. [16] propose “a two-layer controller system structure” that includes a lower computer, responsible for motors’ drivers and lower level sensors, and an upper computer, which is the main controller with autonomous navigation functions. The authors consider the real-time characteristic differences of each task, so they choose a microcontroller unit (MCU) and industrial personal computer (IPC) as the system controllers. The AGV uses an A-star algorithm for navigation planning with guiding nodes, without an explicit lane. The proposed vision system comprises a vision localization system using a label, called ArUco label. This label is placed in the ceiling and when “detected, the processor has a positional solution based on the location information and characteristics of the label”.

The work of Xing et al. [17] presents the coordination of two heavy-duty robotic vehicles, each one using a vision-guided system. Each robotic vehicle independently perceives the path, measures its deviations, and controls the driving motors. It presents simulation and experimental results that show that the two tractors present motion coordination, path-tracking, and control efficiency on straight or curved guide paths. Furthermore, the use of two vehicles enhanced the load-carrying capacity considerably. The vision guidance control system, as in [16], includes a Digital Signal Processor (DSP) for image processing and an ARM-based (Advanced RISC (Reduced Instruction Set Computer) Machine) microcontroller for motion control.

Kondákor et al. [18] compare results of a line-following AGV using vision-based line tracking and an infrared line sensor. The vision system, however, presented a computational time four times higher than the line sensor. However, the authors affirm that “the additional computational time is compensated by lots of advantages”, such as “line changes can be detected in advance”, the system “can be used on uneven surfaces”, and “the susceptibility to the vibration of the car is also much lower” compared with the line sensor.

It is possible to mention other possibilities of the camera and line-following approach use, such as the work of Kang et al. [19] that uses the camera to recognize patterns that control the AGV, for example, direction arrows, and stop signs; Gumus, Topaloglu, and Ozcelik [20] that propose line-following robot application in public transportation using industrial sensors; and Mautz and Tilch [21] that provide a survey of optical indoor positioning approaches. The authors claim that “an improved performance of optical systems has triggered image-based positioning methods to become an attractive alternative for applications in industrial metrology and for robot- and pedestrian navigation”; and Zhou and Liu [22] that use QR codes for the AGV navigation system “to reduce the cost and improve the positioning accuracy”. Despite the growing number of references in line-followers’ vision systems development, as far as the authors are concerned, no published papers discuss the influence of the parameters presented here in the image processing stage and the AGV position control loop.

## 3. Materials and Methods

### 3.1. Automated Guided Vehicle with Mecanum Wheels

**Figure 1:**
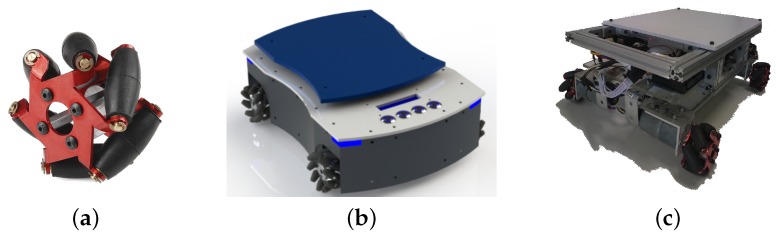
AGV mechanical structure. (**a**) omnidirectional mecanum wheel; (**b**) the projected AGV 3D model with its fairing, Human–Machine Interface (HMI) display, and interface buttons; (**c**) prototype used in this work experiments.

For the experiments, we developed an omnidirectional AGV with mecanum wheels catering to intelligent manufacturing environments. The AGV has four mecanum wheels with a 45º angle. The rotation combination of each of the wheels generates a resultant movement which depends on the speed and direction of rotation of each wheel. This gives the robot the freedom to move in any direction. It has four Pittman 12 V direct current (DC) motors, with a 43.8:1 gear ration, and 0.3 Nm torque. Each motor has an integrated hall sensor quadrature encoder. Figure 1 shows the AGV mechanical structure, while Table 1 shows the dimensions of it.

**Figure 2:**
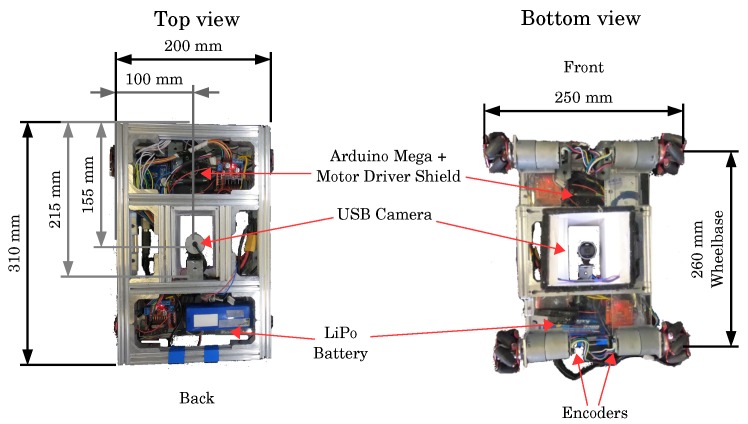
AGV prototype top and bottom views showing its dimensions and components location.

**Figure 3:**
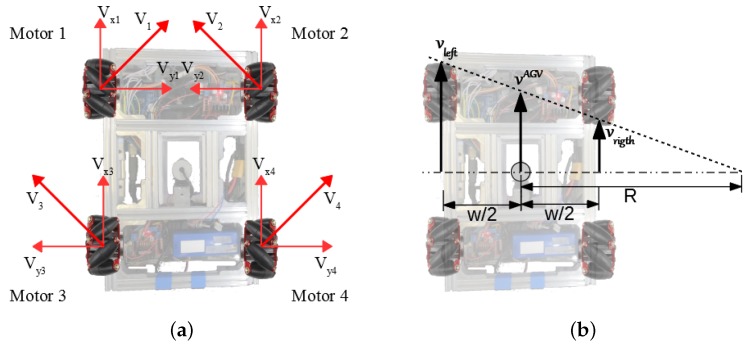
AGV kinematics. (**a**) example of wheels resultant forces applied to the AGV. (**b**) Wheels speed intensity difference.

Figure 2 illustrates the AGV dimensions and components location. With a 260 mm wheelbase, the wheels are located at the ends of the robot. Each motor applies the force of the wheel at a 45° angle to the robot instead of on one of its axes. It creates force vectors in both *x* and *y*-axes. Figure 3a shows that the two front wheels have merging resultant forces and the back wheels have contrary ones.

Despite the holonomic robot configuration, in this paper, we configured the AGV kinematics as a four-wheeled differential drive. The differential-drive kinematics is one of the most common, as shown by the references in Section 2. In Figure 3b, R means the radius of AGV turning from the AGV center and *w* is the distance between the wheels. AGV kinematics follow [23] modeling. Making the velocities $V_{1}=V_{3}$ and $V_{2}=V_{4}$, in Figure 3a, is the same as locating a resultant speed vector on the AGV middle axis, as shown in Figure 3b. Thus, $v_{left}$ is the vector sum of $V_{1}$ and $V_{3}$ and $v_{right}$ is the vector sum of $V_{2}$ and $V_{4}$.

The AGV has programmed simplified inverse kinematics. It bases this block on a constant linear base speed, and the variations in the left and right velocities caused the robot to rotate. On moving forward, both sides have the same linear base speed. As the sensor measures an angle/distance error, the PID (Proportional Integral Derivative) controller calculates the speed difference required to correct the error. For example, in Figure 3b, with $v_{left}$ and $v_{right}$ intensities difference, the AGV is turning right. The Arduino interprets the signal, adding on one side and discounting on the other, and commanding each wheel individually.

### 3.2. Control Systems

**Figure 4:**
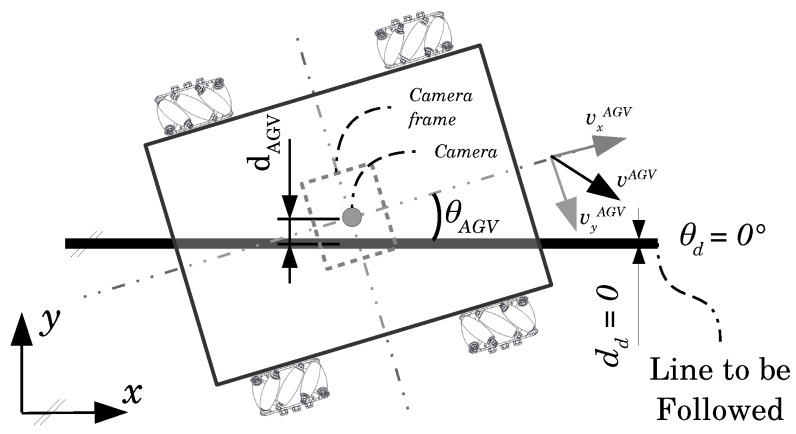
Line following problem formulation.

Consider the AGV in Figure 4. It shows an AGV with a visual guidance system scheme. The camera—whose center matches the AGV center—acquires an image frame capturing the passive guideline position related to the AGV. An image processing algorithm measures the AGV angle $\theta_{AGV}$ and distance $d_{AGV}$ from the line. The desired angle $\theta_{d}$ between the AGV center and the guideline to be followed is equal to zero. In the same way, $d_{d}$ denotes the desired distance from the AGV center to the line, which is also equal to zero. Thus, the problem is with manipulating the wheels’ direction and speed, therefore manipulating $v^{AGV}$, so $\theta_{AGV}$ and $d_{AGV}$ tend to zero.

**Figure 5:**
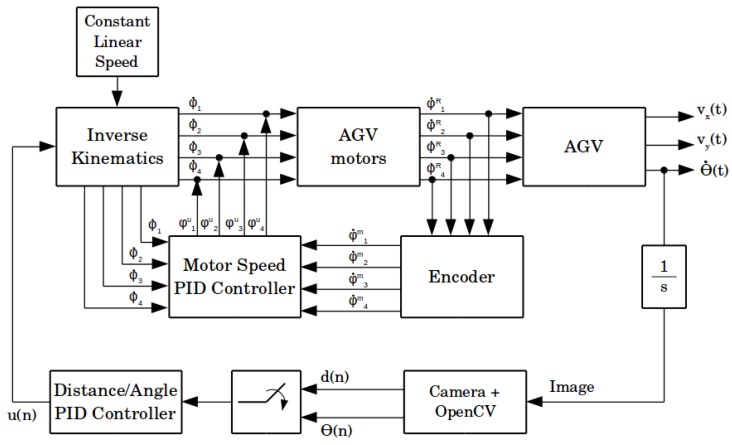
AGV control loop block diagram.

Figure 5 shows the proposed control loop for the line-following problem described. As AGV output signals, we have *x* and *y*-axis velocities, $v_{x}\left(t\right)$ and $v_{y}\left(t\right)$, and the angle deviation $\dot{\theta }\left(t\right)$. A camera acquires an image and, after processing, it is possible to measure the angle $\theta_{AGV}$ and the distance $d_{AGV}$. The PID controller block has one of these two values as input and outputs a control signal. As desired values are both zero, that is, the set-point is zero, then the measured value is the system error.

During operation, the control system set a constant linear speed to AGV, and a control signal input corrected its speed to follow the line. The inverse kinematics block converted the input signals, the linear base speed and the control signal u(n)—which is a speed difference correction—to distinct values ${\dot{\phi }}_{n}$ of speed and direction, with *n* meaning the number of DC motors. These signals triggered the motors, through the power drivers, generating ${\dot{\phi }}_{n}^{R}$ speeds and moving the AGV.

An outer loop is the image processing loop, controlling the AGV trajectory. The inner loop monitored each motor speed. An encoder counted the shaft revolutions, which is possible in order to convert to ${\dot{\phi }}_{n}^{m}$ measured speeds. Through the PID Controller block, the reference signal ${\dot{\phi }}_{n}$ and the measured signal ${\dot{\phi }}_{n}^{m}$ were compared, generating control signals ${\dot{\phi }}_{n}^{u}$ to correct the velocity of each motor. The PID controller is not the focus of this paper. It is a tool, so the AGV followed the line, allowing the sensor to capture the scene and calculate the measures of interest. A more systematic gain tuning could improve its performance, or it points to the use of another control paradigm. This work looks to the sensor performance by now.

**Figure 6:**
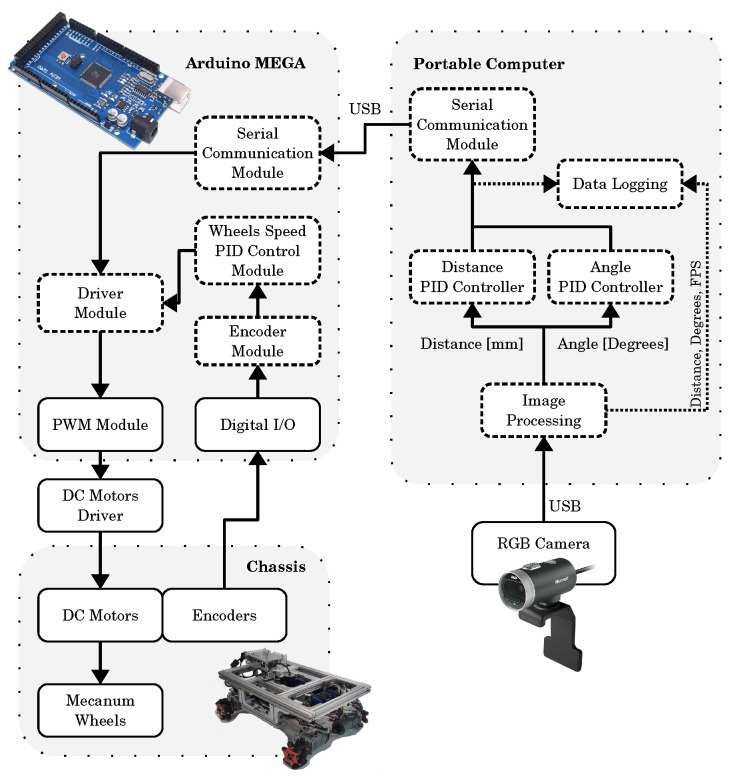
AGV system architecture.

As in [16,17], the system used two processing systems. The higher processor is a portable computer. It performed image acquisition and image processing loops, and the PID controller calculation for deviations on distance and angle from the guide. The lower-level processor is an Arduino Mega 2560, based on the ATmega 2560. It received the linear speed command from the higher-level processor and sent the specific commands for each motor through the DC motor driver. The Arduino is also responsible for reading the encoders and control the individual DC motor speed using a PID strategy. The Arduino board communicates with the higher-level processor by a USB cable and a serial communication protocol. Figure 6 summarizes the architecture.

From the distance/angle offset value measured by the camera, the PID controller calculated the correction value which the higher-level processor sent to the Arduino board. The driver module translated this value into four different speed values for each of the AGV motors, converting a speed value into a PWM (Pulse Width Modulation) command for each of the motor drivers. The lower-level processor also corrected motors speed with a lower-level speed PID controller.

The USB RGB camera is a component of the sensor this work evaluates. The CMOS (Complementary Metal Oxide Semiconductor) sensor is the transducer that interpreted the scene luminous impulses, transforming them into a color information matrix. The webcam itself pre-processed this information before sending it via USB cable. However, this information matrix still needed data processing to extract the AGV angle and distance from the reference line information. Thus, the camera as a position sensor was a block that encompassed the camera itself and all the image and data processing required to extract the scene information of interest. Table 2 describes the specifications of Microsoft LifeCam Cinema webcam used in this work. The camera used a CMOS image sensor, although the manufacturer does not provide information on the model. Because of the application characteristics, the low distance of the fixed focus is an important feature. Despite the undisclosed pixel size, from the field of view angle and a simplified model, we estimate the size of the pixel in Section 3.3.

### 3.3. Line Detection and Measurement Algorithm

As the goal of this paper is to evaluate and analyze the USB camera as a line sensor, the image processing algorithm is a key part of the sensor. However, the line detection and measurement algorithm is the simplest it can be, so it enables parameters variation while keeping a stable control loop.

**Figure 7:**
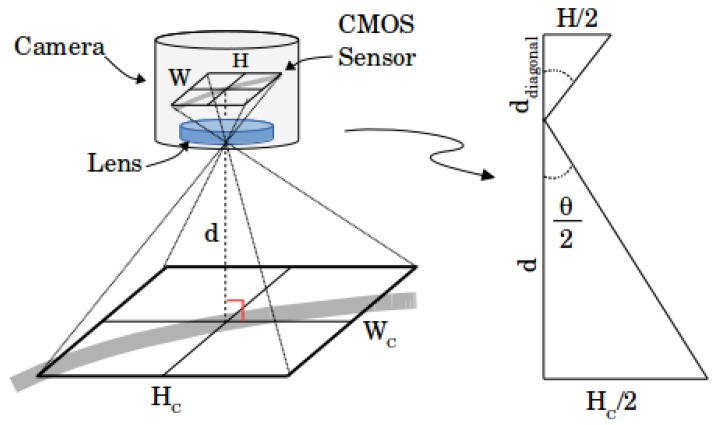
Scheme presenting the camera distance from the surface and the image formation at the CMOS sensor.

The algorithm’s first step is image acquisition. To measure distance and angle, it is important to know the correspondence of the image with the captured surface. Figure 7 presents a simplified model of a camera image acquisition, detailing the camera components, and relation of real scene distances with image distances.

According to [24], if the real length, in meters, of a superficial diagonal captured, $h_{r}$ and its value in image pixels, $h_{diag}$, are known, one then also knows the length of each pixel, $d_{p}$, in meters. Equation (1) shows this first conclusion, but also an approximation to determine $d_{p}$:

(1) $$d_{p}=\frac{h_{r}}{h_{diag}}\approx \frac{2d\tan(\frac{\theta }{2})}{\sqrt{W^{2}+H^{2}}}.$$

Considering the fixed camera distance from the ground, *d*, the image width *W* and height H, and the field of view angle, θ, $d_{p}$ follows from Equation (1). Figure 7 exemplifies the camera image acquisition scheme and shows a simplification with the triangle similarity principle, where $H_{C}$ and $W_{C}$ are the height and the width of camera view surface, respectively.

Table 3 presents the values of Equation (1) variables and the result, $d_{p}$. The Gaussian Pyramid function determines image size, width and length; Table 2 gives the field of view angle θ, and the AGV project defined the distance of the camera from the floor. In addition, Table 3 shows the sensor resolution, i.e., the smallest change the sensor detects. The sensor distance resolution is equal to $d_{p}$, and the angle resolution is 0.6 degrees per pixel, meaning the smallest difference perceived in line angle. Section 3.3.2 explains the angle measurement. In addition, Equation (3) displays the resolution if the difference in the fraction numerator is equal to 1.

The next step of the algorithm is to eliminate noise and pre-process the image to posterior information extraction. This paper used python OpenCV library [25] on code implementation.

#### 3.3.1. Image Noise Elimination Techniques

**Figure 8:**
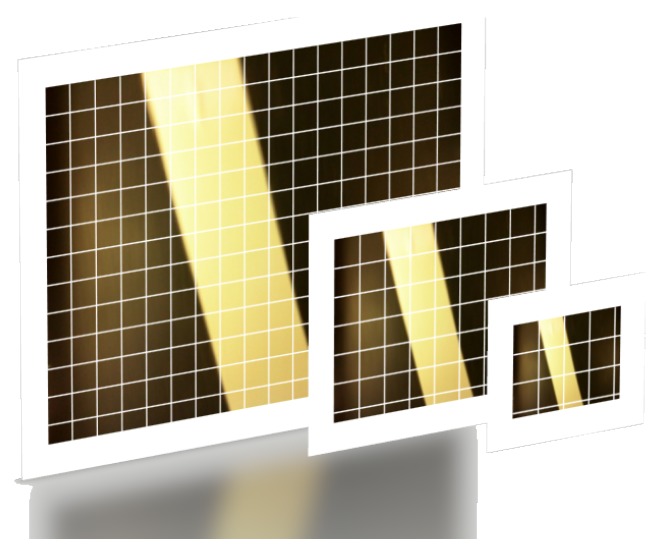
An example of the Gaussian Pyramid downsampling. The higher resolution is the first level, and each new level is half of the previous one.

The camera captured a high-resolution image in terms of the amount of data the AGV control system needs. However, to find the vehicle angle, and distance to the reference line, it was possible to use a downsampled image without a significant loss of information. In this paper, we used the Gaussian Pyramid function to downsample images as the line angle does not depend on image resolution. Although this function is a standard image downsample function, as shown as an example in Figure 8, we used it also to eliminate small high-frequency noises while we still had no loss of relevant information. As the first step in the image processing procedure, Gaussian Pyramid function decreases the computational cost, which impacts the AGV control loop.

After the downsample, the image processing algorithm smooths the image to remove salt-and-pepper noise. This filter takes the median of all pixels under a structuring element (also called a kernel) area and replaces the central pixel with its median value. The larger the kernel, the smoother the result. The median filter kernel is square-shaped, and a positive odd integer defines its size. The OpenCV median filter function replicates the borders to fit the kernel size and to compute boundary pixels output. Trucco and Verri [26] defined an algorithm to the median filter, described in Algorithm 1.

**Algorithm 1:** Median Filter Algorithm according to Trucco and Verri [26].

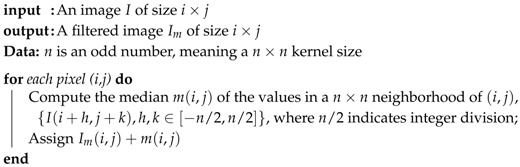



**Figure 9:**
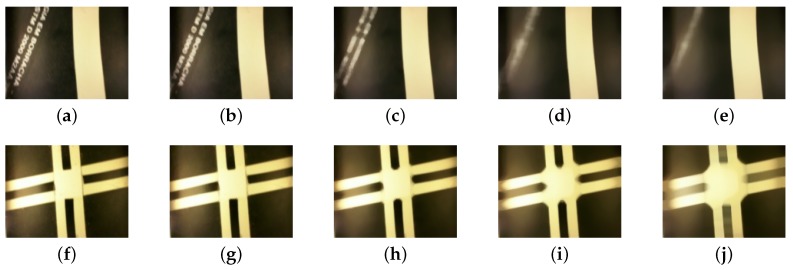
The effect of increasing kernel size in median filter. The first row, images from (**a**) to (**e**), show the filter effect on the reference line with some noise around. The second row presents the effect on the lane crossing. (**a**,**f**) original image without filtering; (**b**,**g**) kernel size: 3×3; (**c**,**h**) kernel size: 7×7; (**d**,**i**) kernel size: 15×15; (**e**,**j**) kernel size: 21×21.

Figure 9 presents the variation of the kernel size of a median filter in two different situations: a single line, and a crossing. On color images, the function executes the median filter at each RGB-channel.

Henceforth, the median filter kernel size is 7×7. As Figure 9i,j show, there is an increasing crossing distortion with the increase of the kernel. The benefit of a median filter in the paper application is the smoothness of noise and the line edges. Moreover, the 7×7 size is the limit of distortion to application image characteristics.

Besides the above techniques, we used a morphological transformation to eliminate larger noise areas in the image, for instance because of lighting reflex, and also to deal with the track crossing. Our algorithm used an opening morphological transformation, which is the dilation of the erosion of an image. An opening is an algebraic operation on an image through a structuring element or kernel. This operation eliminates small islands, smooths contours, and breaks narrow chink smaller than the kernel size. Thus, the impact on the filtered image depends on the structuring element shape and size.

**Figure 10:**
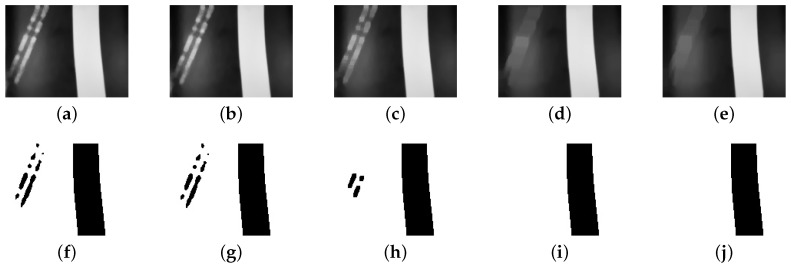
Comparison of different kernel sizes and its impact on noise elimination. The first row shows gray-scale blurred images with distinct opening operations: (**a**) no opening operation; (**b**) kernel size: 2×2; (**c**) kernel size: 5×5; (**d**) kernel size: 10×10; (**e**) kernel size: 15×10. The second row shows, respectively, images after a fixed threshold binarization.

Usually, morphological operations deal with binary images, using Boolean operations. However, it still runs in gray-scale images, through the use of min and max operations. While gray-scale dilation outputs a pixel with the maximum value found over the kernel neighborhood, the gray-scale erosion outputs a pixel with the minimum value. Thus, the gray-scale opening operation assigns to a pixel the maximum of the minimum value on the neighborhood defined by the kernel. Figure 10 compares different kernel sizes application in the same image. The image includes a reference line and some white noise. As the kernel grows, it removes more of the noise and homogenizes the image before the binarization.

**Figure 11:**
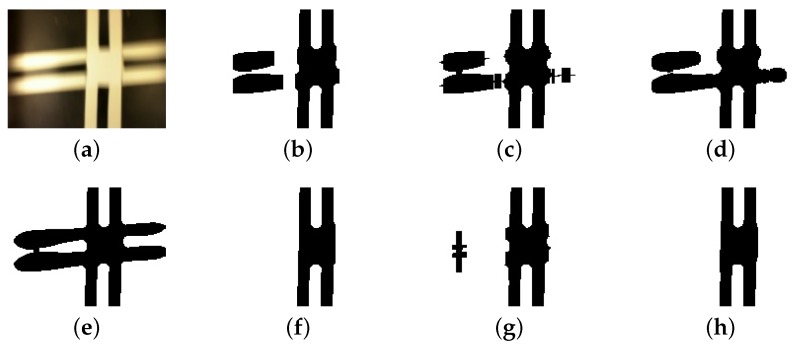
Comparison of different kernel shapes and sizes and its impact on crossing noise elimination. (**a**) original image; (**b**) rectangular Kernel size: 15×10; (**c**) cross-shaped Kernel size: 15×10; (**d**) elliptical Kernel size: 15×10; (**e**) original image binarization; (**f**) rectangular Kernel size: 30×10; (**g**) cross-shaped Kernel size: 30×10; (**h**) elliptical Kernel size: 30×10.

Together with external noise elimination, the opening operation filtrates the horizontal line of the track crossing. Only the vertical information is important and the horizontal line is a noise to the sensor. Thus, using different kernel shapes and sizes, the resultant image shows merely a vertical line. Figure 11 presents the original image and its simple binarization and the results of a kernel variation.

Section 4 investigates the impact of kernel shape, and kernel size of opening operation, as well the image resolution variation on AGV control loop.

As shown in Figure 10 and Figure 11, despite the filters used in the algorithm, the goal is to output a binary image with a clear black line in a white background, increasing the contrast between the object of interest, the tape, and the track floor. Thresholding is one of the simplest and most widely used segmentation techniques. The approach is a pixel operation, making input pixels above a certain threshold level white and others black. On the output image, the line color is black while the real line is white. Thus, the thresholding operation in this work is an inverse operation. Let *k* be a suitable threshold value between 0 (black) and 255 (white), the gray-scale range, and Equation (2) gives the thresholded image $I_{b}$ of size (i×j) from the input image *I* of size i×j.

(2) $$I_{m}\left(i,j\right)=\left\{\begin{array}{cc} 0, & \mathrm{if}\;I(i,j)\geq k,\\ 255, & \mathrm{otherwise}. \end{array}\right.$$

#### 3.3.2. Angle and Distance Measurement

From the binarized image, Algorithm 2 describes the reference line angle measure and Algorithm 3 describes the distance measure from the reference line center. The angle measurement is a basic trigonometric relation.

**Algorithm 2:** Reference line angle determination.

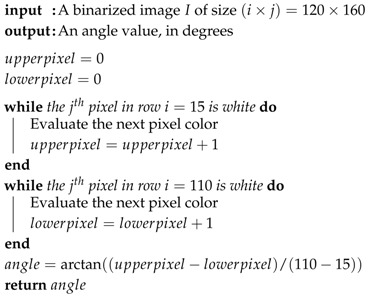



**Algorithm 3:** Reference line distance from center determination.

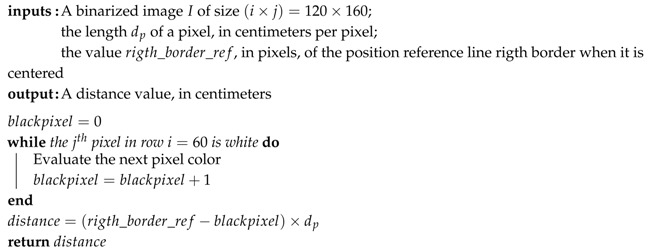



**Figure 12:**
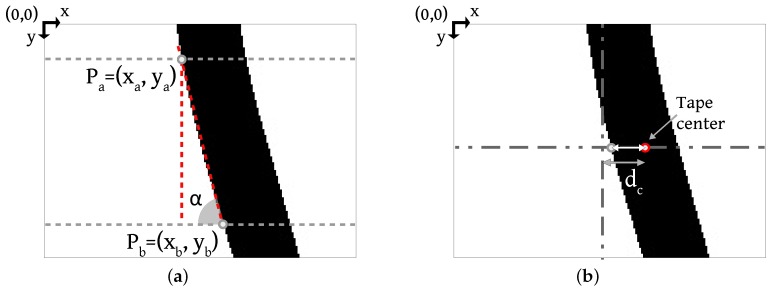
Angle and Distance Measurement. (**a**) the angle between points $P_{a}$ and $P_{b}$; (**b**) the distance of the border of the tape from the image center, matching the desired tape center.

Referring to Figure 12a, the algorithm looks for the first black pixel in two image rows. As we know the points $P_{a}=\left(x_{a},y_{a}\right)$ and $P_{b}=\left(x_{b},y_{b}\right)$, applying Equation (3), we have the angle α related to the reference line center. The vertical line is the zero reference.

In Equation (3), the denominator absolute value guarantees that the angle varies as follows: an AGV rotation from the line center to the right side makes the angle increase positively, while a rotation from the center to the left side makes the angle increase negatively.

To the distance measure, the reference point is the image center, on both the *x* and *y*-axis. At the image central row, the algorithm searches for the first black pixel. Considering the tape width, the measured distance is the tape right border distance from the image center. The algorithm compensates the tape width, as shown in Figure 12b, so the distance $d_{c}$ means the line center distance from the image center. As the reference line approaches the right border of the image, the distance values increase negatively. Otherwise, it increases positively:

(3) $$\alpha =\tan^{-1}\left(\frac{x_{a}-x_{b}}{|y_{a}-y_{b}|}\right).$$

**Figure 13:**
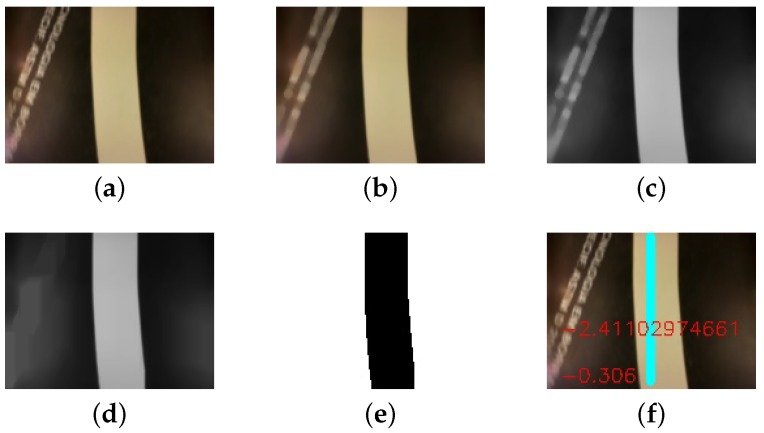
The algorithm resultant images: (**a**) original image; (**b**) after a smoothing filter; (**c**) converted to gray-scale; (**d**) after the opening morphological operation; (**e**) binarized image; (**f**) the original image along a center line, in light blue, and angle and distance errors, respectively.

To summarize, Figure 13 brings the algorithm steps by showing its resultant images. Figure 13a shows the original colored image re-sized by the Gaussian Pyramid and in Figure 13b the image after the median filter, resulting in a smoother image. After this, it converts the image to a gray-scale image, in Figure 13c. Subsequently, it applies the opening morphological operation, resulting in the image on Figure 13d. The binarization of Figure 13d creates the image in Figure 13e. From this image, Algorithms 2 and 3 detect the line and measure its angle and distance related to the reference line. Finally, Figure 13f shows the result of the sensor measures and the control loop receives the errors values. All the steps must occur in a short constant period, so they do not affect the controller.

### 3.4. Definition of Validation Indicators

For the experiments data analysis, we defined four criteria: the mean, the standard deviation, and the range of both angle and distance measurements; and the frames per second rate. From the data mean, we expect to observe the data central tendency. In this paper, the mean is the angle variation average and the average distance from the line during the experiments. Thus, the closest to zero the mean, the better. Although even a mean equal to zero implies the AGV followed the line with a minimum angle and distance variations.

To verify how data vary, we use standard deviation and the range. The first reveals the data dispersion level, i.e., how far from the mean are the samples. The latter is a measure of data spread from the difference between the maximum and the minimum value. Together with the mean, these criteria allow a better analysis. For example, if all values are close to zero, then the AGV followed the line with minor disturbance. However, even with a nonzero mean, the sensor measurements may have significance.

A standard deviation and an amplitude value close to zero mean the system and/or the sensor have a deviation tendency. However, this deviation is persistent. On the camera as a position sensor measurements evaluation, even if the sensor does not present accuracy, we still estimated the measurements exactness in relation to the reference line.

Finally, the frame per second (fps) rate criteria reflect on image processing time and control loop interval. The higher the fps rate, the better. In terms of image processing, it means that the sensor can deliver angle and distance measures with a high frequency, enabling a stable control loop. In the experiments, we used the four criteria to assess AGV response over (a) a variation on morphological operation parameters, using different kernel shapes and sizes, (b) AGV speed increasing, and (c) the increasing of image resolution.

At the first point, the AGV behavior observation with the filter parameters variation aims to appraise the impact of this variation on the controller in the sense of keeping its stability or leading it to instability—in other words, how much the parameter variation can approach the system to instability. The AGV speed change aims to test how much the processing time can affect the controller in case it needs faster answers, i.e., with increasing speed. The AGV speed change aims to test how much the processing time can affect the controller in case it needs faster answers, i.e., with increasing speed. Image resolution is directly proportional to the processing time required to extract the angle and distance information. The aim of varying image resolution is to evaluate the trade-off between the information amount the image contains and the processing time required to extract the interest information. For the line-following problem, since the image has a great contrast between the floor and the object of interest, it does not need the capture of small details.

## 4. Experimentation

**Figure 14:**
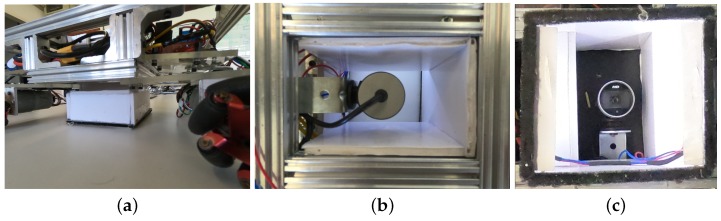
Camera lighting box. (**a**) a lateral view of the box; (**b**) box top view; (**c**) view from the bottom. The LEDs strips are at the image apex and bottom, pointing upwards.

Lighting interference is a major problem that image processing faces. For the first tests, the AGV camera had no forced lighting, having only natural light and laboratory lamps as track lighting. However, interference in line identification has led to the use of a forced lighting apparatus in the robot. Thus, a white box enveloped the camera, sealing it from the external interference, counting on two white LED strips illuminating the lane indirectly. Figure 14 shows the camera box.

As a commercial camera, Microsoft LifeCam has several convenient image features for the average custom, as described in the last line of Table 3. Two relevant features for this article application are auto-focus and auto-exposure. By default, these features are active and create the automatic focus and illumination correction of the image. However, when using the camera in the AGV, the features are more harmful than useful. The focus variance during AGV operation changes the tape width. Even momentarily, it distorts the angle and distance calculation. Likewise, auto-exposure causes variations in the camera shutter aperture as the robot moves and scene illumination varies, causing the line and floor tonality change. At a low exposure degree, the algorithm may not detect the tape, while, at a high exposure degree, the tape and part of the floor may be identified as the reference line.

**Figure 15:**
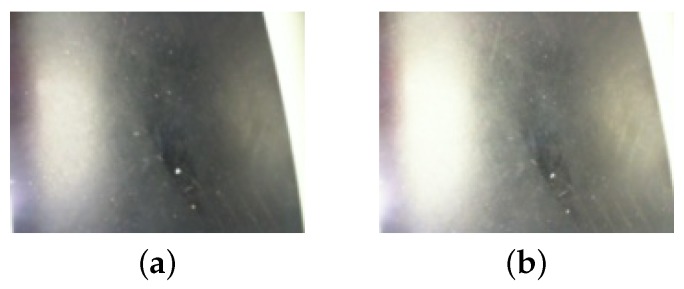
An example of image variation with default settings. (**a**) image of a scene before auto-exposure correction; (**b**) the same scene after auto-exposure correction.

In this way, we customized the camera’s operation options. In its final setting, the automatic focus and exposure compensation are shut down. Absolute focus and absolute exposure are constant values. In addition, the color temperature was also changed, correcting the white balance. Figure 15 shows the effect of auto-exposure feature. The aperture correction expands the light reflex, making it brighter. It causes an image misinterpretation.

**Figure 16:**
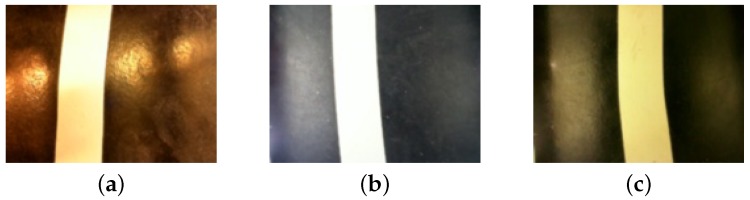
Comparison among the images on different camera settings. (**a**) no forced lighting box and camera default settings; (**b**) AGV with the forced lighting box and camera default settings; (**c**) AGV with the forced lighting box and camera custom settings.

Figure 16 compares the AGV camera settings. In (a), the AGV has no forced lighting box and the camera settings are the default. In (b), the AGV has a forced lighting box, but the camera settings are the default. Note the saturation on the tape color caused by the auto exposure. In (c), the AGV has a forced lighting box and the camera settings are customized, preserving original colors.

**Figure 17:**
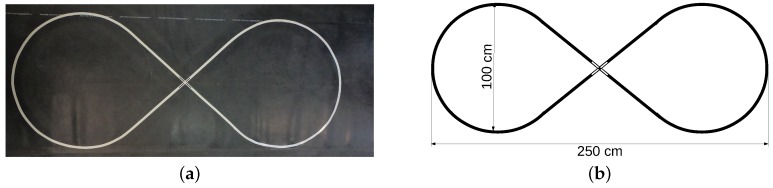
Eight-shaped lane used in experimental trials. (**a**) the real lane; (**b**) lane schematic.

Figure 6 describes the AGV used in the experiments, and Table 4 describes the hardware and software specifications. The camera and the Arduino MEGA board connect to the computer via USB cables. The experimental environment is an eight-shaped lane with 100 cm wide by 250 cm long. The lane floor is an ASTM D2000 natural rubber and the reference line is a 19 mm gray vinyl electrical tape. Figure 17a shows the real experimental setting in the laboratory. To reproduce a condition closest to the industrial floor condition, people could step on the track. Figure 17b shows the lane schematic.

**Figure 18:**
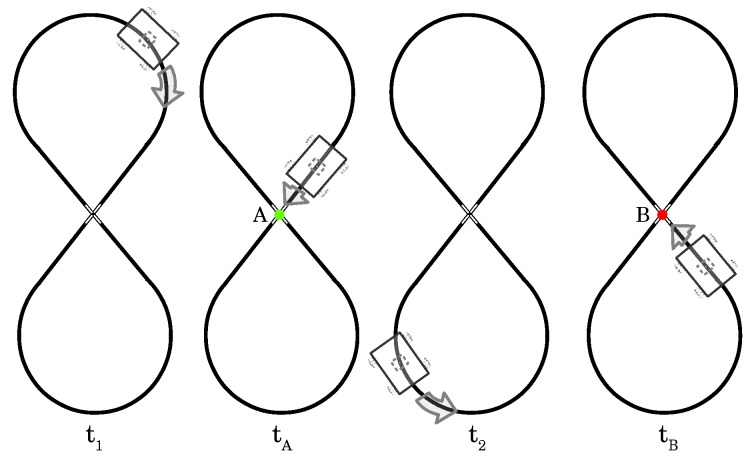
AGV trajectory and its reference points to data analysis.

The lane shape implies a crossing. AGV image processing approaches it like noise and eliminates it in both directions, as shown in Figure 11. To clarify the data analysis, we consider the crossing as a reference point depending on the AGV direction. Referring to Figure 18, at instant $t_{1}$, AGV is in a right turn approaching the lane crossing, in $t_{A}$. In this direction, we name it the crossing point A. In the same way, at instant $t_{2}$, AGV is turning left and, in $t_{B}$, it approaches the crossing, and we name it crossing point B. The sequence of three crossing points means the robot completed a cycle, a full lane trajectory.

### 4.1. Kernel Shape and Size Variation

**Figure 19:**
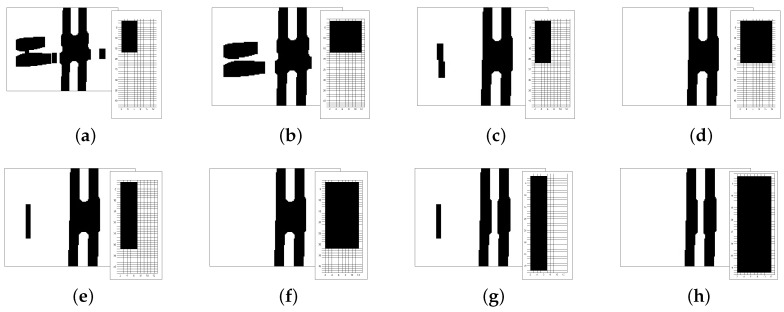
The effect of increasing the size of the rectangular kernel. (**a**) size 15×05; (**b**) size 15×10; (**c**) size 20×05; (**d**) size 20×10; (**e**) size 30×05; (**f**) size 30×10; (**g**) size 40×05; (**h**) size 40×10.

The first experiment comprises the structuring element of the opening morphological operation shape and size variation. For each structuring element, the AGV takes several laps on the lane, registering the instant it crosses points A and B. We use three distinct shapes, varying each one in two sizes. Rectangular, elliptical and cross-shapes are the OpenCV kernels shapes for the experiment. Initially, the kernel size was defined according to the need to eliminate noise at the lane crossing point. We tested the rectangular-shaped kernel in an extensive range of values. Then, the best values were used in the other formats for comparison. For example, Figure 19 presents the effect of kernel increasing in the binarized image. As the kernel size grows, the lower the noise is around the reference line. The number of columns produces more change than the number of rows, in this case, as one can see comparing from Figure 19c to Figure 19h. On the other hand, as one can see in Figure 19g,h, increasing the kernel meets a limit when it impacts the object of interest.

**Figure 20:**
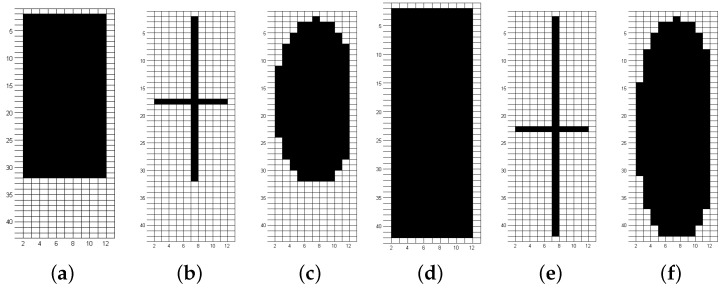
The structuring elements used um Opening operation. (**a**) rectangular, size 30×10; (**b**) cross-shaped, size 30×10; (**c**) elliptical, size 30×10; (**d**) rectangular, size 40×10; (**e**) cross-shaped, size 40×10; (**f**) elliptical, size 40×10.

From the initial experiments, analyzing the noise rejection, the next experiments standard kernel size defined was (30×10) and (40×10). Thus, the shapes and sizes definition results in kernels shown in Figure 20. Figure 11 has already presented images after the morphological operation with each kernel shape.

### 4.2. AGV Speed Variation

The experiment aims to verify how the AGV speed affects the sensor measurements. While the AGV speed varies, the sensors variables are constant, such as fps rate, kernel shape and size, and image resolution. On the speed variation trials, fps rate is 148 frames per second, and the morphological operation uses a rectangular structuring element of size 30×10, on a 160 by 120 resolution image.

As described in Section 3.1, the AGV control system foresees a linear base speed constant. The PID controller alters the speed of each side to turn the AGV and corrects the angle and/or the distance error. For the experiment, we set a range of linear base speeds to measure angle and distance and observe the sensor response.

The hypothesis is that, as the AGV velocity increases, the scene variation is faster, and the sensor still needs to acquire image without motion blur. This effect is the presence of smears around the object and its movement path, like a shadow. Thus, the experiment meets to identify the motion blur occurrence on the speed range from 0.08 m/s to 0.29 m/s, and its impact on measures. For each speed, the AGV takes several laps on the eight-shaped lane, registering the instant it crosses points A and B.

### 4.3. Image Resolution Variation

The original captured image size is 640 by 480 pixels. Usually, with a greater resolution, the image offers more details of the scene. However, for this work application, the scene has a controlled environment with forced lighting, besides presenting a high contrast between the floor and the AGV reference line.

With the resolution variation, the distance per pixel parameter also changes, and must be calculated again. Likewise, the rows’ positions in both angle and distance algorithms change for each resolution, so the resultant measurement is the same. The values were changed proportionally to the resolution increase since we made the initial calculations for the 160 by 120 resolution. Table 5 shows the parameters values at 320 by 240 and 640 by 480 image resolutions.

**Figure 21:**
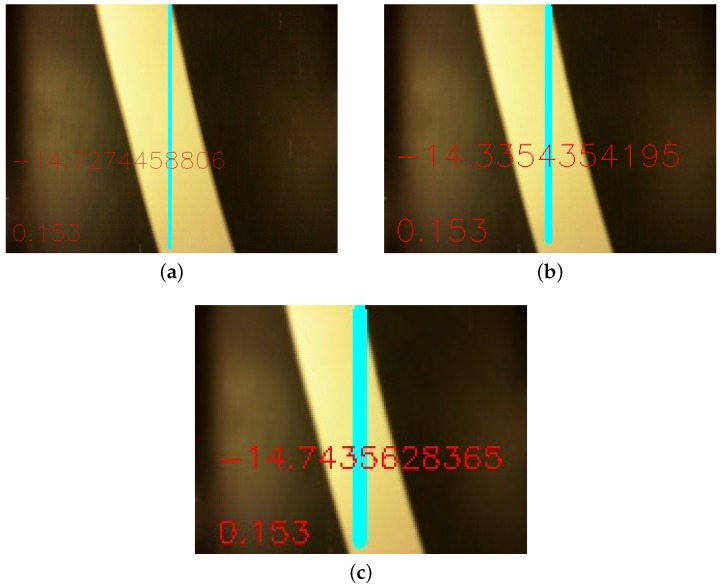
Angle and distance measurements in different resolution. Images do not follow their real sizes. (**a**) size: 640×480. $\theta_{AGV}≊-14.727446^{\circ }$ and $d_{AGV}=0.153$ cm; (**b**) size: 320×240. $\theta_{AGV}≊-14.335435^{\circ }$ and $d_{AGV}=0.153$ cm; (**c**) size: 160×120. $\theta_{AGV}≊-14.743563^{\circ }$ and $d_{AGV}=0.153$ cm.

As expected, the distance per pixel $d_{p}$ is proportional to the image resolution. In addition, Figure 21 indicates the values of angles and distance measured in each of the resolutions, starting from the higher resolution image, and applying the Gaussian Pyramid function successively. Comparing the three images, one can observe the image sharpness decreases as the image resolution decreases. Despite the resolution and sharpness difference, the distance measure continues to be the same. The angle measure has a little deviation. However, comparing Figure 21a to Figure 21c angle measurement, the discrepancy between them is in the hundredths decimal place.

From the AGV control loop point of view, the increase in image resolution means an increase in the time interval required for image processing, which causes an increase in the sensor signal sampling time. Thus, the sensor refresh rate decreases.

For the lowest image resolution, the image processing and the desired values computation time are, on average, 6.5 ms. Doubling the resolution to 320 by 240 pixels raises the same time interval to an average of 12 ms, and another increment to 640 by 480 pixels drives the processing time interval to 38 ms. The time intervals above correspond only to the sensor processing time interval, without considering the control loop processing and the communication between the higher level and lower level processors. In the same way as the previous tests, for each of the three image resolutions, the AGV takes several laps on the eight-shaped lane, registering the instant it crosses points A and B.

### 4.4. Experimental Results

**Figure 22:**
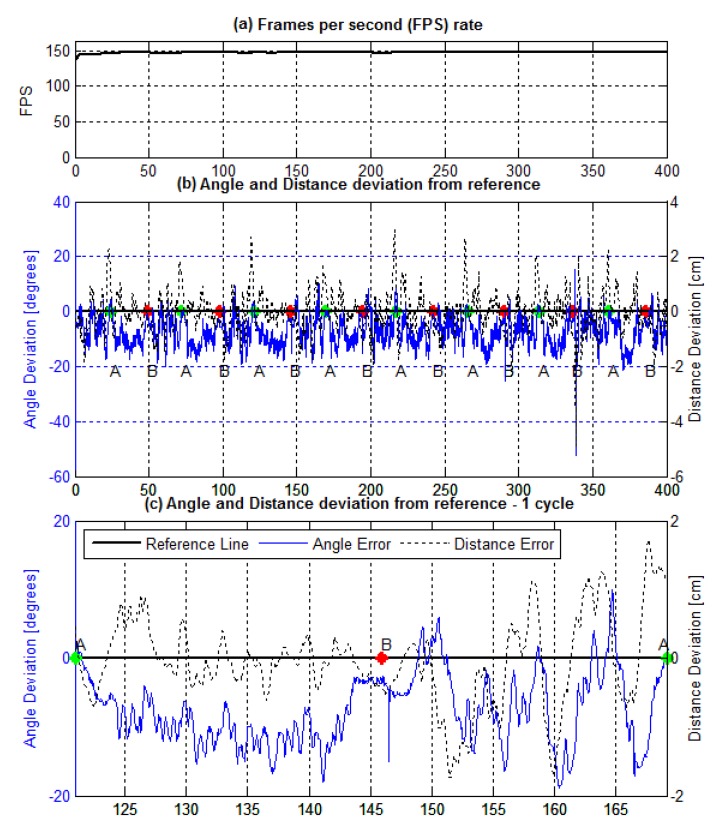
AGV line following the trial on the eight-shaped lane, eight complete laps, with rectangular kernel of 30×10 size. (**a**) frames per second rate during the trial; (**b**) total trial data for angle and distance measurements; (**c**) a single cycle detail.

From all the tests and along the trajectory, the fps was monitored, the angled and distance measurements stored, and the instant that the AGV passes through the lane crossing recorded. Figure 22 shows how we measure the experimental data.

The first graph, Figure 22a, presents the behavior of frames per second rate during the trial. Figure 22b displays the measurements along the trial, eight complete laps. At each crossing point, A or B, the time instant was recorded to guide posterior analysis. In addition, a single lap, or a cycle, is detailed in Figure 22c. For clarity, the next results show a cycle of the course.

On Figure 22c graphs, one can observe a behavior tendency on the AGV locomotion. In the course between points A and B, i.e., when the lane has a left turn, a cumulative angle measurement error holds, while the distance measure continues around the reference line. In contrast, in the circuit from point B towards point A—i.e., a right turn—both angle and distance measurement vary around the reference line, and the distance measure follows the angle one more than in the previous course. Note in Figure 22b B that this behavior repeats during the trail.

As cited, this work aims not to get the best performance of the PID controller, but to evaluate how the sensor parameters variation affects its stability. Being that, the controller performance on Figure 22 results is prone to improvements. Conversely, for the interest measures, the controller and the system stability and performance were sufficient, requiring no extra dedication to PID gains’ fine-tuning.

#### 4.4.1. Kernel Shape and Size Variation

**Figure 23:**
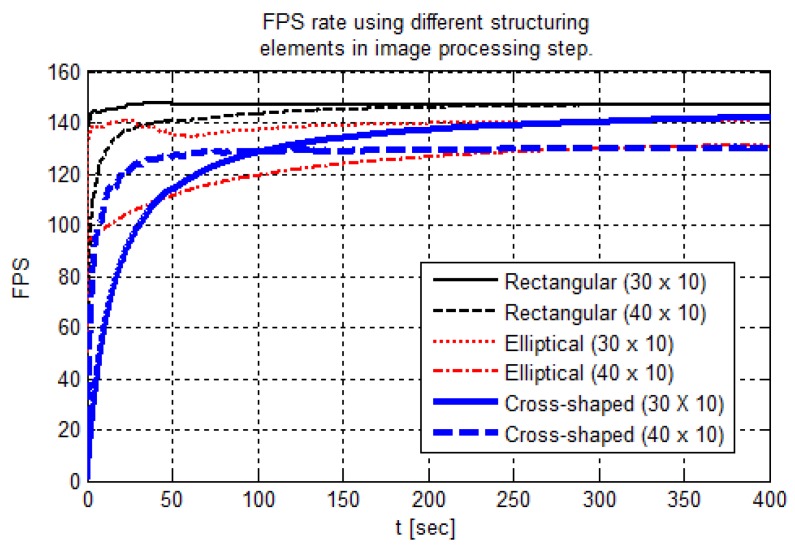
Frames per second rate for different kernel shapes and sizes.

As the results, Figure 23 presents the fps rate behavior for the six different structuring elements used in the morphological operation at the image processing step. Due to the morphological operation characteristics, the kernel attributes changing alters the image processing time.

A bigger pixel neighborhood—i.e., using a bigger structuring element—increases the computation cost. However, only when using elliptical- and cross-shaped kernels is this expected behavior proved. Although the bigger rectangular kernel takes more time to settle, both kernels converged to 148 frames per second rate.

On the other hand, when elliptical- and cross-shaped kernels have the same size, the fps rate converges to closer values. Thus, the kernel size is a processing time determinant factor for the indicated shapes. Nevertheless, the control loop total processing time variation was about 0.8 ms between the best and the worst fps rate. This fps rate level keeps a control time interval of 7 ms, approximately, from the sensor sample to the controller response.

**Figure 24:**
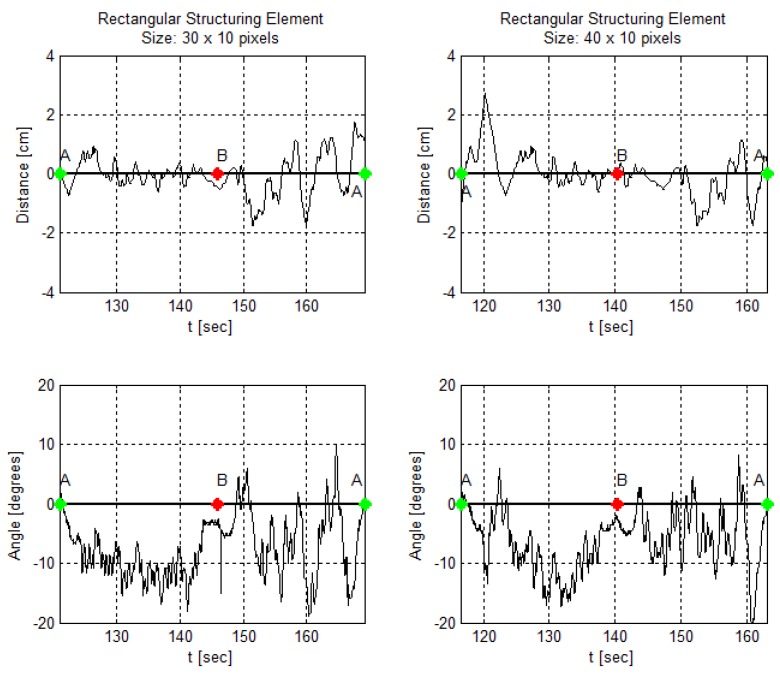
AGV angle and distance measures using a rectangular kernel in the morphological operation at the image processing step.

**Figure 25:**
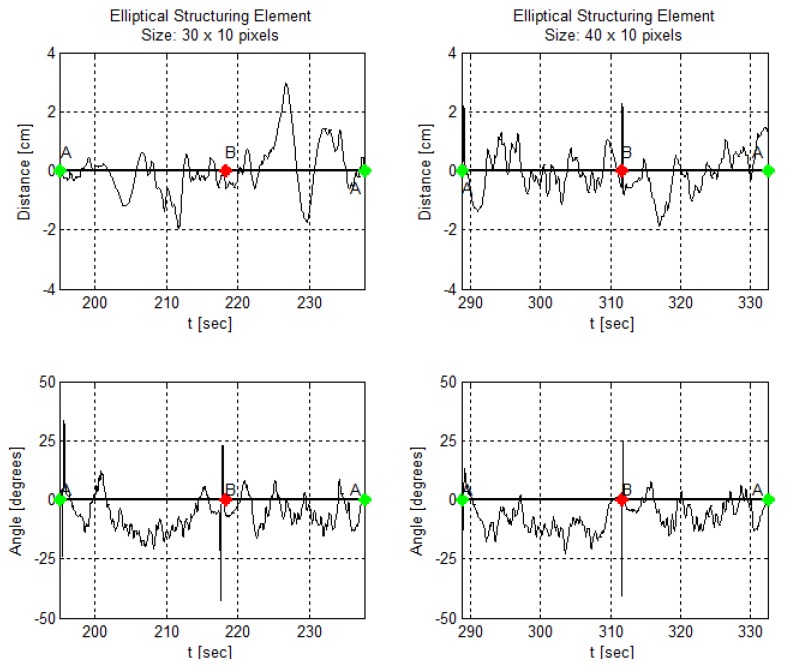
AGV angle and distance measures using an elliptical kernel in the morphological operation at the image processing step.

**Figure 26:**
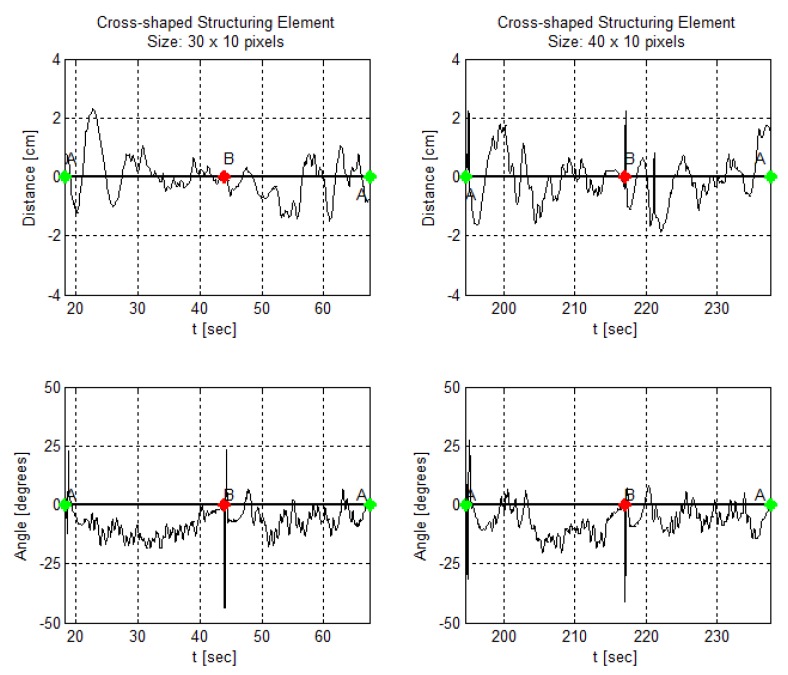
AGV angle and distance measures using a cross-shaped kernel in the morphological operation at the image processing step.

In addition, Figure 24, Figure 25 and Figure 26 demonstrate angle and distance measurements on a lane cycle for each kernel. The kernels’ configurations were tested individually; then, each cycle has a different time interval.

The figures display the lap with the best response in terms of the errors’ mean and standard deviation. An emphasized alteration around the crossing points, A and B, means an inaccurate angle measure. This incorrect measurement occurs if the image filters do not eliminate the lane intersection horizontal line, as shown in Figure 11g, for example. It still does not affect the article interest analysis.

In addition, in Figure 24, Figure 25 and Figure 26, one can, visually, perceive a gradual increase in the distance error, as the kernels vary in shape and size. Likewise, the angle behavior along the trajectory and curves remains the same as identified in Figure 22.

#### 4.4.2. AGV Speed Variation

**Figure 27:**
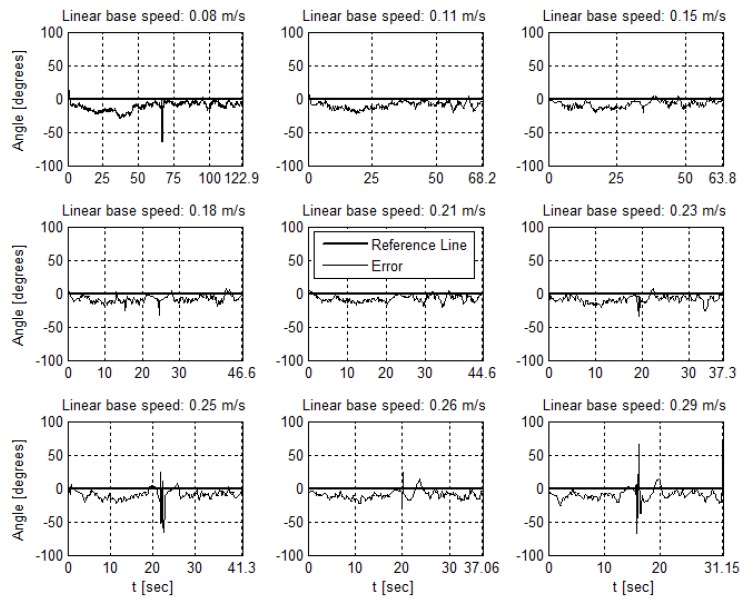
A cycle of angle measurements at each AGV linear base speed.

**Figure 28:**
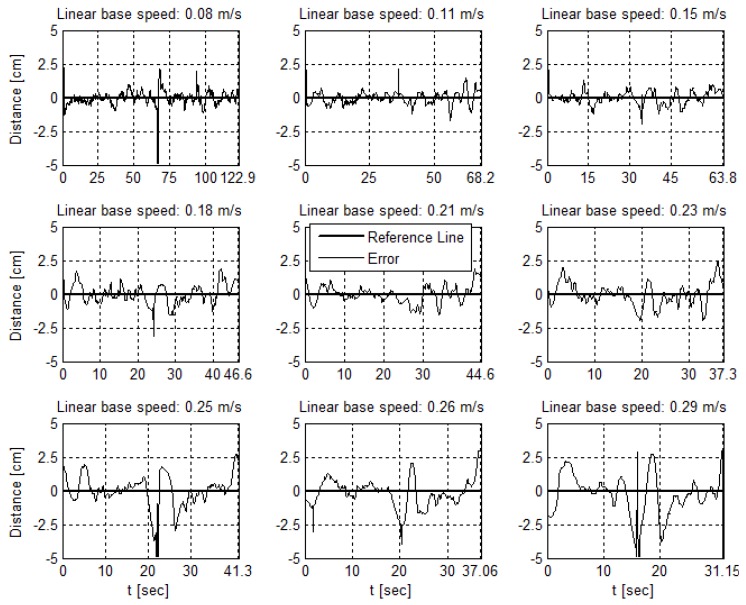
A cycle of distance measurements at each AGV linear base speed.

Figure 27 and Figure 28 present the measurements of the AGV angle and distance from the reference line in a range of linear base speeds. The time elapsed on each cycle increases as the speed increases. The 0.29 m/s base speed drives the controller to instability, since the AGV loses the line before complete a full lap.

The speed variation affects images acquisition, as the scene changes in a higher frequency, but also has an impact on the controller. An AGV speed variation requires a new controller tuning. However, it does not fit the focus of the work. Therefore, the AGV uses the same controller for the speed range described. The impacts on the image and measurements are the relevant aspects. In addition, for all trials, we keep the same image processing settings, using the rectangular kernel, size 30×10. Thus, there was no impact on fps, keeping it close to 150 frames per second.

In addition, the speed change varies the time the AGV takes to complete a lap. Still, the same behavior is observed in terms of angle and distance error. The speed increase influence on the angle measurement, Figure 27, is not as sharp as it is in the distance measurement, Figure 28.

In images analysis, for the speed range used, the camera acquires the images without presenting the motion blur effect. More pronounced angle variations as seen in the velocities 0.25 and 0.29 m/s graphs are due to the noise at the intersection combined with the higher speed of the robot.

#### 4.4.3. Image Resolution Variation

**Figure 29:**
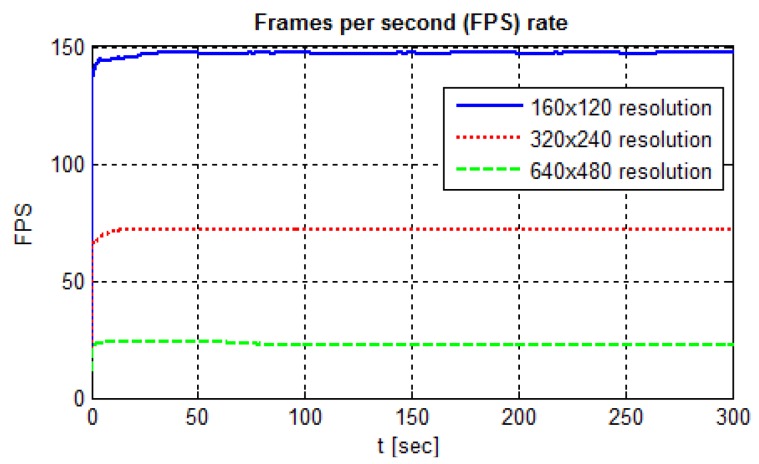
Frames per second rate per image resolution.

Figure 29 presents the results of frames per second rate per image resolution. The increase in resolution of the input image has a direct impact on the fps and the control loop time interval. The computational cost to process higher resolution images enlarges, increasing the time between image reading, processing, and calculation of the measurements, controller actuation and updating the outputs for the actuators. The minimum control interval, i.e., with the maximum fps rate, is about 7 ms, doubling to 14 ms with the intermediate resolution and reaching 45 ms for the maximum resolution.

**Figure 30:**
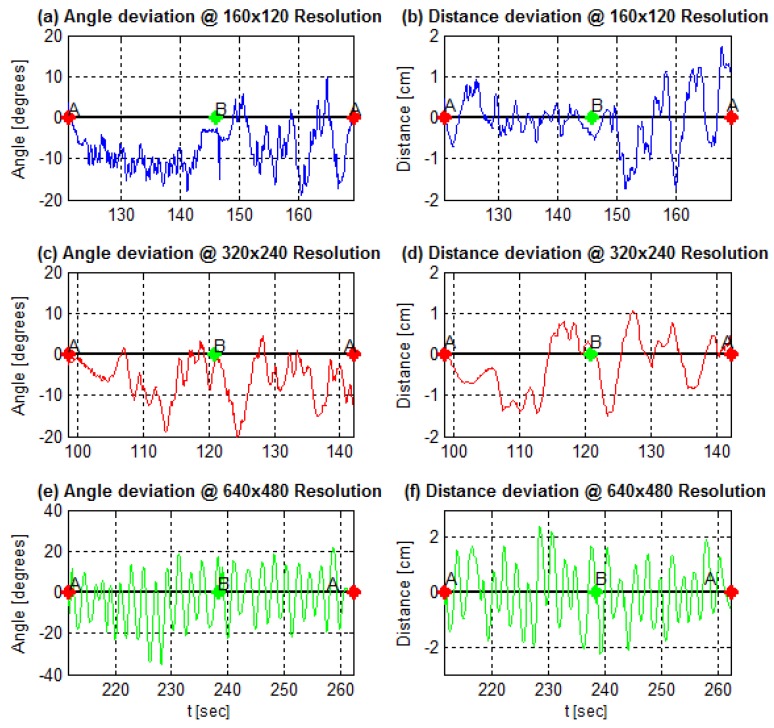
A lane cycle for each image resolution.

Figure 30 displays the measurements of angle and distance from reference line at each resolution. With trials performed separately and as the resolution affects the AGV control loop, the cycles have specific start and end instants.

## 5. Discussion

**Figure 31:**
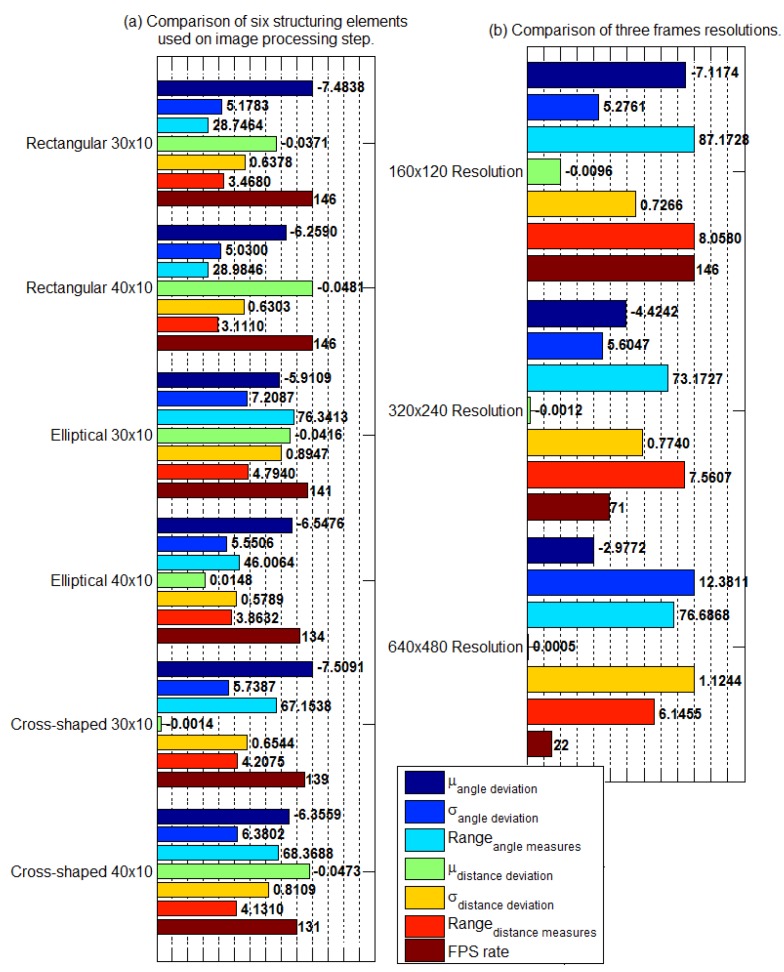
Trials results comparison. (**a**) structuring element, or kernel, variation; (**b**) frame resolution variation.

As a summary of trials results, Figure 31 shows a bar graph for the comparison among kernel characteristic shift results and image resolution results. The charts use the metrics Section 3.4 presents. For clearness, we normalized the values to allow a comparison through the bar chart. At each bar, the left side is the metric value, and one should compare the metrics among those of the same sort. In addition, Table 6, Table 7, Table 8 and Table 9 summarize the results and allow the comparison and the analysis of each parameter variation. To verify the parameter impact in the AGV position control loop, we modify one parameter, while the other two remain fixed. Table 6 summarizes the kernel shape and size variation with a 160 × 120 pixels image resolution and a linear speed of 0.21 m/s. Table 7 summarizes the image resolution change with a fixed kernel and the same linear speed of Table 6. In addition, Table 8 and Table 9 summarize the linear speed variation with the lower image resolution and a rectangular kernel with a fixed size.

Before the sensor results analysis, the classic PID controller used in the AGV performs a simple algebraic sum of angle and distance control actions. It treats each error separately and sums the response, as it is the speed difference correction to the motors. However, the gain tuning of the variables was conflicting. During the tuning tests, as the gains of one variable increase, and reduce its error, the other variable response deteriorates. Thus, the PID controller response in the AGV system points to using a multivariable controller for the line-following problem.

The results’ qualitative analysis of the bar chart in Figure 31a and Table 6 show no specific kernel emerging as the best option. One can observe a trade-off between the angle indicators and the distance indicators’ improvement. The metric of interest determines the kernel result evaluation. Together with the application characteristics, it suggests which is the recommended kernel.

If compared to the others, the rectangular kernels have the best results considering the highest fps rate and the low values of angle and distance ranges. Although the elliptical kernel of size 40×10 presents lower values of distance mean and distance standard deviation, the outcome of the rectangular ones is balanced, between the measured variables. Inspecting the sensor, lower levels of standard deviation and range demonstrate a higher exactness. Thus, the results of kernel shape and size variation indicate that the image processing techniques are determinant in the camera measurement exactness. In addition, the technique modification can contribute or degrade the sensor exactness. Still, from the control system point of view, the image processing technique affects the control loop time interval, making it another variable on choosing the right problem approach.

The image resolution variation results in Figure 31b and Table 7 show a critical impact on the sensor fps rate as the image resolution becomes larger while the other parameters remain the same. In this work, control architecture, showed, in Figure 5, the impact on the fps rate reflecting on the entire control loop. A reduced fps rate of the sensor means a greater control loop delay. Observe in Figure 30e,f the influence of increasing the control loop time interval on system response. This increase in the loop time interval drives the system close to the marginal stability. In addition, in a digital controller, the control loop time interval, or sample time, must remain the same during the operation. The digital PID gains tuning depends on the sample time. Thus, the image resolution variation trials showed for the paper application and for the used hardware that the image resolution is not a flexible variable in the problem. In the sense of the control loop, image resolution variation impacts the PID controller tuning, altering its sample time and its performance. Thus, considering the same scenario—the same application and same hardware—a trade-off between higher measurement resolution and the control interval required for the application is necessary. Still discussing the higher image resolution trial, its results present the lower error average both for angle and distance, the last being close to zero. However, Figure 30e,f and the standard deviation elevated levels for both measurements show dispersed data. This fact reinforces the need for evaluating the sensor metrics jointly.

In the case of Table 8 and Table 9, the linear speed variation impacts image acquisition, but it has major impacts on the PID controller. Since we tuned the controller to the linear speed of 0.21 m/s, its variation deteriorates the controller performance. Values different from 0.21 m/s of the measurement range—both for angle and distance from the line—show a higher variability, or at least are at the same level. The impact on the fps rate is not clear.

The qualitative analysis of the sensor allows concluding that the USB camera is a sensor solution to the line-follower problem. It offers a distance measurement resolution about 0.5 mm for the lower image resolution and about 0.125 mm for the higher image resolution. In relation to the angle resolution, it has about 0.6 degrees for the lower image resolution and can reach 0.16 degrees at the higher one, surpassing the commercial sensors mentioned above.

When dealing with a commercial camera, the embedded pre-processing to the average consumer can affect the use of the equipment in a specific application. Therefore, it is necessary to adapt the camera with customized configurations. Each camera has its attributes, so each one must be tested and configured in your application. As webcams are suitable for generic demands, the higher quality ones offer several automatic features.

In this work case, the automatic adjustment attributes were deactivated and configured according to the characteristics and specifications of the AGV. These attributes, useful in a generic usage, degrade the quality of the image acquired by the camera of the line following AGV. For instance, the auto-focus feature activated during AGV movement and scene variations contributes to variations in the tape width. Despite the decrease in effect after the use of a box with forced lighting, its occurrence still resulted in erroneous measures, even if momentarily. Exposure adjustment caused an aperture variation depending on the AGV position on the lane. The feature, useful for the webcam use in brightness varying situations, provided false lane detection. Consequently, the sensor computes false angle and distance measures in these situations. The purpose of turning off the settings was to minimize the noise caused by the self-tuning.

In this paper’s trials, the sensor reached an fps rate greater than the specified in Table 3. The rate measurement occurred during the control code processing, and we calculated it in two different ways. Despite the need for more inquiries, we highlight two points raised that contribute to the attainment of this rate. One is the use of camera custom settings, which may have contributed to a decrease in the pre-processing required when using automatic settings. The other point is the camera specific use with a lower level code for the image processing instead of an ordinary application such as streaming on the internet. The code collects and processes each frame hastily, without storing it. Thus, we believe that the 30 fps rate described by the manufacturer applies to normal use, in high-level software, with internet streaming, e.g., which consumes much more in terms of processing time. However, by mainly comparing the cost with specific cameras, the equipment proved to be feasible for the application.

## 6. Conclusions

The main objective of the article was to investigate how the image processing required using the USB camera as a position sensor impacts in a line-follower AGV control loop. The paper proposed to evaluate the camera use as a sensor in the AGV position/path control loop and also assess the impact of image resolution, image processing parameters, and frame per second rate on the control response. The implementation relied on a PID controller. The sensor set was composed of a webcam and an image processing code using OpenCV for measuring the line angle and the distance of the AGV from the center of the line.

The paper’s main contribution is a qualitative and quantitative analysis for the different sensor configurations. In addition, the work presents a systematic way of investigating the accuracy of the camera as a position sensor for an AGV and the line following problem. From the methods used, it is possible to trace new tests to verify sensor characteristics and their impact on the AGV control.

The results show that the USB camera can be used as a position sensor, showing a competitive sensor in terms of response time and measurement resolution. The results also suggest the measurement noise main causes and the image processing parameters that influence the sensor response. Variations in image processing techniques may degrade the sensor response, affecting the response of the control loop. For the test scenario and the hardware used, the variation in the image resolution presented the greatest impact on the control loop, driving the system closer to the marginal stability.

As future work, the use of USB camera RGB color channels separately allows an investigation on cases in which the use of one color improves the algorithm processing time, enables robustness over color noise, and enhances the measurement. After testing AGV image processing with different lighting color, processing each color channel individually, we can compare the results with the color image processing.

Likewise, embedding the processing code on a microcomputer in the AGV, such as a Raspberry Pi, is future work and evaluating whether the hardware meets the image processing time needs and the appropriate control time interval for the line-follower problem. From this article’s results, it is necessary to define the processing bottleneck of a microcomputer with a much lower computational capacity for this type of application.

In addition, in terms of control, the work points in two directions. The first point concerns the controller performance. As discussed, there is a trade-off regarding the gain tuning of the PID controller of this work. It suggests the use of a multivariate controller. In addition, the AGV behavior, i.e., the response differences depending on the curve direction, denote system nonlinearities and lead to the use of an adaptive controller. The other direction refers to the sensor. It brings measurement uncertainties and a measurement variation in the presence of noise. Thus, the use of a robust controller for this problem must be evaluated. In this sense, we plan to investigate the relationship between the proposed sensor and the controller response in the future. The work should verify the impact of the sensor parameters’ variation in terms of position control evaluation, positioning accuracy level, and AGV oscillation and vibration during load transport.

## Figures and Tables

**Table 1:** Mecanum AGV dimensions and specifications.

Description	Quantity
AGV Body Length	310 mm
AGV Body Width	200 mm
AGV Body Height	130 mm
Wheel Diameter	54 mm
Max. Linear Velocity of the AGV Body	0.58 m/s
Mass of AGV Body	3.4 kg

**Table 2:** Parameters description of the cameras used in the experiments.

Features	Microsoft LifeCam Cinema Model: H5D-00013
Still Resolution	5 Megapixels
Video Modes	1280 × 720 pixels video
Lens	Wide-angle lens
Sensor Technology\ Model	CMOS image sensor\ Not disclosed
Fixed Focus	from ∼0.15 m to infinity
Field of View	73° diagonal field of view
Max. Frame rate	30 fps
Interface	USB 2.0
Image Features	Digital pan, tilt, and 4x digital zoom; Auto focus; Automatic image adjustment with manual override.

**Table 3:** Image variables and pixel size equivalency.

Description	Quantity
*d*	6.8 cm
Width x Length	160×120 pixels
θ	73°
$d_{p}$	approx. 0.051 cm/pixel
angle resolution	approx. 0.603°/pixel

**Table 4:** Hardware and software description.

**Processor**	Intel^®^ Core^™^ i7-3630QM CPU @ 2.40 GHz x 8
**Graphics**	Intel^®^ Ivybridge Mobile
**RAM memory**	4 GB
**Operational System**	Ubuntu 16.04 LTS
**Python version**	2.7.12
**OpenCV library**	3.3.1

**Table 5:** Image variables and pixel size equivalency for greater resolutions.

Description	Quantity		Description	Quantity
*d*	6.8 cm		*d*	6.8 cm
(Width×Length)	640×480 pixels		(Width×Length)	320×240 pixels
θ	73°		θ	73°
$d_{p}^{640\times 480}$	approx. 0.0125 cm/pixel		$d_{p}^{320\times 240}$	approx. 0.0255 cm/pixel
Angle resolution	approx. 0.163°/pixel		Angle resolution	approx. 0.318°/pixel

**Table 6:** Kernel shape and size variation results summary with a 160×120 pixels image resolution, and a linear speed of 0.21 m/s.

Kernel Shape	Rectangular		Elliptical		Cross-Shaped	
**Kernel Size [pixels]**	30×10	40×10	30×10	40×10	30×10	40×10
**Angle Deviation Mean**	−7.4838	−6.259	−5.9109	−6.5476	−7.5091	−6.3559
**Angle Deviation Standard Deviation**	5.1783	5.03	7.2087	5.5506	5.7387	6.3802
**Angle Measurements Range [degrees]**	28.7464	28.9846	76.3413	46.0064	67.1538	68.3688
**Distance Deviation Mean**	−0.0371	−0.0481	−0.0416	0.0148	−0.0014	−0.0473
**Distance Deviation Standard Deviation**	0.6378	0.6303	0.8947	0.5789	0.6544	0.8109
**Distance Measurements Range [cm]**	3.468	3.111	4.794	3.8632	4.2075	4.131
**fps rate**	146	146	141	134	139	131

**Table 7:** Image resolution variation results summary with a 30×10 pixels size rectangular kernel, and a linear speed of 0.21m/s.

Image Resolution [pixels]	160×120	320×240	640×480
**Angle Deviation Mean**	−7.1174	−4.4242	−2.9772
**Angle Deviation Standard Deviation**	5.2761	5.6047	12.3811
**Angle Measurements Range [degrees]**	87.1728	73.1727	76.6868
**Distance Deviation Mean**	−0.0096	−0.0012	−0.0005
**Distance Deviation Standard Deviation**	0.7266	0.774	1.1244
**Distance Measurements Range [cm]**	8.058	7.5607	6.1455
**fps rate**	146	71	22

**Table 8:** Linear speed variation results summary with a 160×120 pixels image resolution, and a 30×10 pixels size rectangular kernel. Speed values lower than 0.21 m/s.

Linear Speed [m/s]	0.08	0.11	0.15	0.18	0.21
**Angle Deviation Mean**	−9.5431	−9.2255	−8.2625	−8.0161	−7.4838
**Angle Deviation Standard Deviation**	7.6585	5.1106	4.9409	5.2089	5.1783
**Angle Measurements Range [degrees]**	88.1462	28.2526	28.1629	42.7565	28.7464
**Distance Deviation Mean**	−0.0342	−0.0357	−0.0527	−0.0666	−0.0371
**Distance Deviation Standard Deviation**	0.6886	0.4713	0.5179	0.7159	0.6378
**Distance Measurements Range [cm]**	7.1655	4.335	4.029	5.7502	3.468
**fps rate**	130	136	124	130	146

**Table 9:** Linear speed variation results summary with a 160×120 pixels image resolution, and a 30×10 pixels size rectangular kernel. Speed values greater than 0.21 m/s.

Linear Speed [m/s]	0.21	0.23	0.25	0.26	0.29
**Angle Deviation Mean**	−7.4838	−9.1616	−7.9728	−8.388	−8.6504
**Angle Deviation Standard Deviation**	5.1783	5.9414	7.4547	7.8457	10.185
**Angle Measurements Range [degrees]**	28.7464	56.1869	90.0964	95.2142	134.9394
**Distance Deviation Mean**	−0.0371	−0.0408	−0.0257	−0.7328	−0.5598
**Distance Deviation Standard Deviation**	0.6378	0.8898	1.1358	1.838	1.8675
**Distance Measurements Range [cm]**	3.468	7.599	7.854	7.854	7.8349
**fps rate**	146	129	139	148	136

## Abbreviations

The following abbreviations are used in this manuscript:

AGV	Automated Guided Vehicles
RGB	Red–Green–Blue
RFID	Radio Frequency Identification
AGC	Automated Guided Cart
LiDAR	Light Detection and Ranging
QR Code	Quick Response Code
ARM	Advanced RISC Machine
RISC	Reduced Instruction Set Computer
PID	Proportional Integral Derivative
PWM	Pulse Width Modulation
fps	Frames per Second
